# Genetic analysis of the *FBXO42* gene in Chinese Han patients with Parkinson’s disease

**DOI:** 10.1186/1471-2377-13-125

**Published:** 2013-09-25

**Authors:** Kai Gao, Xiong Deng, Wen Zheng, Zhi Song, Anding Zhu, Xiaofei Xiu, Hao Deng

**Affiliations:** 1 Center for Experimental Medicine, the Third Xiangya Hospital, Central South University, 138 Tongzipo Road, Changsha, Hunan 410013, China; 2 Department of Neurology, the Third Xiangya Hospital, Central South University, Changsha, China

**Keywords:** Parkinson’s disease, *FBXO42*, *FBXO7*, Variants, Haplotype

## Abstract

**Background:**

Parkinson’s disease (PD), the second most common neurodegenerative disease, is characterized by loss of dopaminergic neurons in the substantia nigra. The clinical manifestations of PD encompass a variety of motor and non-motor symptoms. Mutations in the F-box protein 7 gene (*FBXO7*) have been identified to cause Parkinsonian-pyramidal syndrome, an autosomal recessive form of Parkinsonism. The F-box protein 42 gene (*FBXO42*), a paralog of the *FBXO7* gene, is involved in the ubiquitin-proteasome system that may play a role in the pathogenesis of PD.

**Methods:**

To determine whether the *FBXO42* gene is associated with PD, we performed a systematic genetic analysis of the *FBXO42* gene in 316 PD patients and 295 gender-, age-, and ethnicity-matched normal controls.

**Results:**

We identified a novel variant c.1407T>C (p.S469S) and three known single nucleotide variants, including rs2273311, rs12069239 and rs35196193 in the *FBXO42* gene in PD patient group. None of the three known variants displayed statistically significant difference in either genotypic or allelic distributions between patient and control groups (all *P* > 0.05). Haplotype analysis showed that a common haplotype (G-C-G) for the three single nucleotide variants conferred a 1.69-fold increased risk for PD (*P* = 0.008 after Bonferroni correction, OR = 1.69, 95% CI = 1.06-2.71).

**Conclusions:**

Our findings suggest that a haplotype of the *FBXO42* gene might be associated with a higher susceptibility to PD.

## Background

Parkinson’s disease (PD) is the second most common neurodegenerative disorder after Alzheimer’s disease. The prevalence of PD increases with age, from about 1% among individuals over age 65 to about 4% in those over age 85 [1]. It is characterized clinically by motor manifestations, including bradykinesia, resting tremor, rigidity and postural instability [2]. However, increasing evidence indicates that non-motor manifestations are common during the course of the disease, and become particularly disabling in advanced stages of PD [3]. Though environmental factors have been identified to increase PD risk, accumulating evidence suggests that genetic predisposition contributes to the pathogenesis of the disorder as well [4]. In the last two decades, at least 18 chromosomal loci (PARK1-18) have been assigned to PD through classic linkage analysis or genome-wide association studies [5], and eight genes (*SNCA*, *LRRK2*, *Parkin*, *PINK1*, *DJ*-*1*, *ATP13A2*, *VPS35* and *EIF4G1*) have been linked to autosomal PD [6]. Mutations in genes responsible for monogenic forms of PD have also been identified in some sporadic cases of PD. These findings support the hypothesis that genetic factors may be implicated in both familial and sporadic PD [7]. Though a number of susceptibility variants for sporadic PD have been identified by genome-wide association studies, the association between some of the variants and PD is unable to be replicated due to a variety of factors, including racial differences, insufficient power, population stratification and differences in sample size [8]. The genetic causes in majority of sporadic cases of PD remain unknown, suggesting that other genetic variations may also contribute to the development of the disease.

Mutations in the F-box protein 7 gene (*FBXO7*), encoding a protein of the F-box protein family, have been identified as a cause for Parkinsonian-pyramidal syndrome, an autosomal recessive neurodegenerative disease with severe levodopa-responsive Parkinsonism, and additional pyramidal signs [9]. Recent evidence from genetics and animal model has suggested a possible role of the *FBXO7* gene in PD [10,11].

The *FBXO42* gene, a paralog of the *FBXO7* gene, encodes another important member of the F-box protein family. It is known that paralogs often retain similar function [12] and may play similar roles in the development of a certain disorder [13]. Additionally, FBXO42 is involved in protein degradation via the ubiquitin-proteasome system that is proposed as a potential mechanism for PD [14-16]. The aim of the present study is to determine whether the *FBXO42* gene is associated with PD in Chinese Han population.

## Methods

### Patients and controls

Three hundred and sixteen unrelated Chinese Han patients with PD (Male/female = 160/156; age years 61.68 ± 11.20; onset age years 58.53 ± 12.37), and 295 gender-, age- and ethnicity-matched healthy controls (Male/female = 150/145; age years 62.91 ± 11.45) without any family history of neurological disorders were recruited for this study. All patients were tested at Department of Neurology, the Third Xiangya Hospital of Central South University and diagnosed according to accepted diagnostic criteria [2]. The control subjects were recruited from the Third Xiangya Hospital Medical Center and a standard clinical neurological examination was performed on all control subjects to exclude a diagnosis of possible idiopathic PD. There was no statistically significant difference in age or gender between patient and control groups (*P* > 0.05, using χ^2^ test for gender and the Student’s t-test for age). The protocol of this study was approved by the Ethics Committee of the Third Xiangya Hospital of Central South University and all the individuals signed informed consent.

### Genetic analysis

Genomic DNA (gDNA) was isolated from lymphocytes using standard phenol-chloroform method. Polymerase chain reaction (PCR) was carried out in a reaction volume of 25 μl, containing 100 ng of gDNA and 10 pmol of each primer, in the 9700 Thermal Cycler System (Applied Biosystems Inc, Foster City, CA). The PCR consisted of 35 cycles of denaturation at 95°C for 40 s, annealing at 58°C for 35 s, and extension at 72°C for 40 s, and a final extension step at 72°C for 5 minutes. PCR amplified all coding region and intron/exon boundaries of the *FBXO42* gene by using 14 primer pairs (Additional file 1: Table S1, available online) and a two-step screening strategy was performed in this study. In the first step, mutation in the coding region and flanking sequence of the *FBXO42* gene was screened by previously described method in 151 PD patients (Male/female = 77/74; age years 61.84 ± 12.75; onset age years 58.14 ± 14.38) [17]. In the second step, the risk of the variants was evaluated between enlarged PD group (316 patients including first 151 patients) and gender-, age- and ethnicity-matched normal controls (295 individuals) to increase statistical sensitivity. A sequenced normal control and a negative control (without DNA sample) were set in every experiment. The abnormal single strand conformation polymorphism bands of PCR products were sequenced using ABI 3500 genetic analyzer (Applied Biosystems Inc, Foster City, CA).

### Statistical analysis

The power of the study was calculated using Power and Sample Size Program [18]. The power to detect association with the disorder in 316 cases and 295 controls was estimated to be 83.7%, 80.2%, and 81.0%, with a relative risk of ≥1.6 at a significance level of 0.05 when testing variants with the minor allele frequencies of 0.409, 0.298, and 0.039, respectively. All the variants were tested for deviation from Hardy–Weinberg equilibrium (HWE). Association analysis was carried out using chi-squared test or fisher’s exact test to assess genotypic or allelic association between PD and each of the variants. Haplotype analysis, a molecular genetic testing to identify a set of closely linked segments of DNA that used in linkage analysis or when a given trait is in linkage disquilibrium with a marker or set of markers, was also performed to estimate the association of a haplotype with PD. Haplotypes with a frequency less than 0.03 were excluded in the analysis and Bonferroni correction was applied for all significant *P* values. Statistical analysis was performed using PASW18.0 (SPSS Inc., Chicago, IL, USA) and PLINK 1.07 (http://pngu.mgh.harvard.edu/~purcell/plink/). A value (*P* < 0.05, two-tailed) was considered to be significant.

## Results

A novel variant c.1407T>C was identified in a 78-year-old male patient with sporadic PD and was absent in all controls (Additional file 2: Figure S1, available online). The single nucleotide variation does not change amino acid (p.S469S) or splicing (predicted by http://www.fruitfly.org/seq_tools/splice.html). Three known single nucleotide variants, including c.15G>A (p.S5S, rs2273311), c.1411C>G (p.P471A, rs12069239), and c.1525G>A (p.A509T, rs35196193) were found in our PD cohort. None of the three known variants showed deviations from HWE in control group, or patient group, or the cohort as a whole. None of the three known variants displayed statistically significant difference in either genotypic or allelic distributions between patient and control groups (all *P* > 0.05, Table 1). Multivariable haplotype-based analysis indicated that a haplotype, G-C-G, might increase the risk for PD (*P* = 0.002, OR = 1.69, 95% CI = 1.06-2.71). The association between the haplotype and PD remained to be statistically significant after Bonferroni correction (*P* = 0.008, Table 2).

**Table 1:** **Genotypic and allelic distributions of *FBXO42 *variants in PD patients and control subjects**

**Variants**	**Amino acid change**	**Genotype/Allele**	**N (%)**		***P*-value (χ**^**2**^**)**	***P*-value (χ**^**2**^**)**	**OR (95% CI)**
			**Cases**	**Controls**			
rs2273311	p.S5S	GG	31 (9.81)	26 (8.81)			
		GA	138 (43.67)	126 (42.71)			
		AA	147 (46.52)	143 (48.48)	0.85 (0.32)		
		G	200 (31.65)	178 (30.17)			
		A	432 (68.35)	412 (69.83)		0.58 (0.31)	1.07 (0.84-1.37)
rs12069239	p.P471A	CC	219 (69.30)	191 (64.75)			
		CG	92 (29.11)	93 (31.53)			
		GG	5 (1.59)	11 (3.72)	0.18 (3.45)		
		C	530 (83.86)	475 (81.51)			
		G	102 (16.14)	115 (19.49)		0.13 (2.35)	1.26 (0.94-1.69)
rs35196193	p.A509T	GG	294 (93.04)	268 (90.85)			
		GA	20 (6.33)	27 (9.15)			
		AA	2 (0.63)	0 (0.00)	0.16 (3.15)		
		G	608 (96.20)	563 (95.42)			
		A	24 (3.80)	27 (4.58)		0.50 (0.46)	1.22 (0.69-2.13)

OR, Odds ratio; CI, confidence interval.

**Table 2:** **Haplotype analysis of rs2273311-rs12069239-rs35196193 in the *FBXO42 *gene in PD patients and controls**

**Haplotype**	**Frequency in cases**	**Frequency in controls**	***P*-value (χ**^**2**^**)**	***P***_***corr***_	**OR (95% CI)**
G-G-A	0.0353	0.0459	0.35 (0.88)	0.47	0.72 (0.32-1.62)
G-G-G	0.1145	0.1468	0.10 (2.78)	0.19	0.75 (0.47-1.21)
G-C-G	0.1710	0.1099	**0.002 (9.31)**	**0.008**	**1.69 (1.06-2.71)**
A-C-G	0.6792	0.6975	0.49 (0.47)	0.49	0.92 (0.65-1.30)

*P*_*corr*_: *P*-value after Bonferroni correction; OR: odds ratio; CI, confidence interval; statistically significant results are marked in bold.

## Discussion

PD is the most common neurodegenerative cause of Parkinsonism, a neurological syndrome characterized by lesions in the basal ganglia, especially in the substantia nigra. Mutations in the *FBXO7* gene have been identified to cause Parkinsonian-pyramidal syndrome, an autosomal recessive Parkinsonism with pyramidal tract signs [9]. We hypothesize the *FBXO42* gene, a paralog of the *FBXO7* gene, as a potential candidate gene for PD because paralogs often retain similar functions [12].

The *FBXO42* gene, mapped on chromosome 1p36.13, contains 11 exons and spans about 105 kb. It encodes a 717-amino-acid protein characterized by an approximately 40-amino-acid F-box motif in its N-terminus and 3 central kelch repeats downstream of the F-box [19,20]. FBXO42 is associated with Skp1, Cul1, and Rbx1, and may function via assembly of an SCF complex [19]. The SCF complex is the largest E3 ubiquitin ligase family that promotes the ubiquitin-dependent degradation of various regulatory proteins, thus controlling various biological processes, including cell cycle progression, gene transcription, signal transduction, and DNA replication [21]. It has been shown that *FBXO42* is transcriptionally regulated by p53, a tumor suppressor playing an important role in regulating the cell cycle and triggering apoptosis [22]. FBOX42 forms an auto-regulatory negative feedback loop with p53 to promote ubiquitination and degradation of p53 [19,23]. *In vitro* studies have found that inhibition of p53 prevents 6-hydroxydopamine-induced cell loss [24,25]. *In vivo* animal studies suggest that *p53* knockout mice or mice pretreated with a p53 inhibitor are protected from dopaminergic neuron death in the pars compacta of substantia nigra [26-28]. Additionally, several studies have also indicated that p53 is involved in the pathogenesis of PD through *alpha*-*synuclein*, *Parkin* and *DJ*-*1*[29-31], mutations of which are known causes for PD phenotype [32].

In the present study, we screened the entire coding region and intron/exon boundaries of the *FBXO42* gene in 316 patients with sporadic PD and 295 gender-, age-, and ethnicity-matched controls. Four variants were identified, including a novel one, and three known single nucleotide variants. The novel variant, c.1407T>C, was detected in a 78-year-old male patient only. However, this variant does not change amino acid (p.S469S) or alter splicing, suggesting the variant is unlikely a pathogenic mutation. For the three known variants, rs2273311, rs12069239 and rs35196193, there was no significant difference between patient and control groups for allelic or genotypic distributions (all *P* > 0.05). Our results suggest that none of the four variants identified in the coding region of the *FBXO42* gene seem to play a major genetic role in the development of PD in Chinese Han population. Larger studies are needed to confirm our findings.

Single-marker association analysis is sometimes not sufficient in complex diseases. The haplotype-based linkage disequilibrium mapping has become a powerful and robust method for genetic association studies, especially in search of complex disease-causing genes [33,34]. Our data indicated that a common haplotype G-C-G (rs2273311-rs12069239-rs35196193) of the *FBXO42* gene conferred a 1.69-fold increased risk for PD. The remaining question is which haplotype is the key player in the development of PD.

## Conclusions

To our knowledge, this is the first study to evaluate the *FBXO42* gene in a cohort of PD patients and controls. Data from the present study suggest that the variants in the coding region of the *FBXO42* gene may play little or no genetic role in PD, but a common haplotype in the *FBXO42* gene may contribute to the susceptibility to PD in this Chinese Han population. More studies with a larger sample size from diverse races are warranted to confirm the results of our findings.

## Abbreviations

ATP13A2: ATPase type 13A2 gene; CI: Confidence interval; EIF4G1: Eukaryotic translation initiation factor 4-gamma 1 gene; FBXO7: F-box protein 7 gene; FBXO42: F-box protein 42 gene; LRRK2: Leucine-rich repeat kinase 2 gene; OR: Odds ratio; PCR: Polymerase chain reaction; PD: Parkinson’s disease; PINK1: PTEN-induced putative kinase 1 gene; SNCA: α-synuclein gene; VPS35: Vacuolar protein sorting 35 gene.

## Competing interests

The authors declare that they have no competing interests.

## Authors’ contributions

KG performed the genotyping, statistical analysis, and drafted the manuscript. XD, WZ, ZS, AZ, and XX contributed to the collection of materials, participated in the study design and coordination, and drafted the manuscript. HD conceived the study, participated in its conceptual design and coordination, and revised the manuscript. All authors read and approved the final manuscript.

## Pre-publication history

The pre-publication history for this paper can be accessed here:

http://www.biomedcentral.com/1471-2377/13/125/prepub

## Supplementary Material

Additional file 1: Table S1 Primers for the *FBXO42* gene. Click here for file

Additional file 2: Figure S1 Sequencing analysis of the *FBXO42* gene. (A) The arrow shows the normal sequence. (B) The arrow shows c.1407T>C (p.S469S) nucleotide substitution. Click here for file
