# Supplementary material for: Genetic analysis of the FBXO42 gene in Chinese Han patients with Parkinson’s disease
Source: BMC Neurol. 2013 Sep 25;13:125. doi: 10.1186/1471-2377-13-125 (PMC3848964; doi:10.1186/1471-2377-13-125)
Supplement: Additional file 1: Table S1 — Primers for the FBXO42 gene. [file 1471-2377-13-125-S1.doc]

**Table S1 Primers for the *FBXO42* gene**

| **Fragment** | **Forward primer (5’→3’)** | **Reverse primer (5’→3’)** | **Product size ( bp )** |
| --- | --- | --- | --- |
| 1 | TGAGCTGCTTTCAGTGGAATACTG | TCCTGATACGGTGAGAGAAAGGACA | 265 |
| 2 | CCAGTATTGGAGGCTGAGGAGA | CCGGGTAGTATCGTCTAATGCT | 285 |
| 3 | AGTGTCTGGACTGAAGGGTTTGC | TCTGGAAATATGACCACAGGCAGG | 208 |
| 4 | GGGCCTCAGAATTTTCACAGCA | TTCCATGCCAAGGGAGTCACC | 238 |
| 5 | CGGATTGTTGTGAGATCCTGAATGC | CCACTGATTTAAGGCCTTTTCTTCC | 262 |
| 6 | TGGTGCTGACTTCTTGACTTCCTCC | GGCACCTGGTCCCTAATTCACT | 240 |
| 7 | GTCCCTGGGAGCTCCTAACT | AGACGCTACAGTGTTTGCTGA | 191 |
| 8 | TCAGAAACCTGTGTCTTTGTAGG | GCCATGGAAATAGTCTTGTATACTC | 211 |
| 9 | TCACACCTGAGTCTTGTTATTGG | CCTGACAAAGTAATGTGGTTTTCC | 226 |
| 10 | GTGTGAGCACTGGTCCTTTGT | CGTCTCCTCTGGCTGGGGAA | 293 |
| 11 | GCAGACTCCTTCAGGTTCCCG | GGGATTACTACTGGAAGCGGGT | 292 |
| 12 | CCGACGAGGATCACTACCAGA | GAAGACCCAAGAGGAGGACT | 300 |
| 13 | ATGTCCTCCAAAGGCCCCTCG | CCGCCCCTTCTCCTTGGTGT | 282 |
| 14 | CCAGAGTATGAACTGCAAGCCCA | AAGCCACAGAAAAGGAAAGGGGT | 280 |
