# Supplementary figures and images for: Genetic analysis of the FBXO42 gene in Chinese Han patients with Parkinson’s disease
Source: BMC Neurol. 2013 Sep 25;13:125. doi: 10.1186/1471-2377-13-125 (PMC3848964; doi:10.1186/1471-2377-13-125)

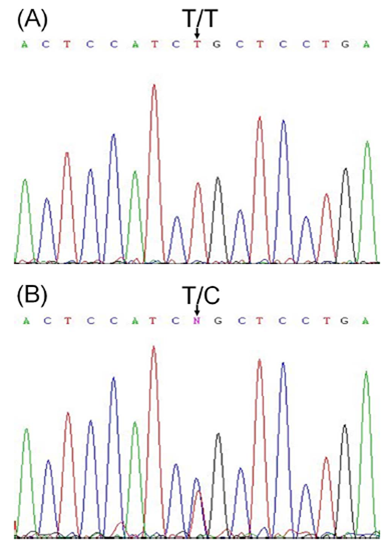

Supplement: Additional file 2: Figure S1 — Sequencing analysis of the FBXO42 gene. (A) The arrow shows the normal sequence. (B) The arrow shows c.1407T>C (p.S469S) nucleotide substitution. [file 1471-2377-13-125-S2.tiff]
